# Black and White men younger than 50 years of age demonstrate similar outcomes after radical prostatectomy

**DOI:** 10.1186/1471-2490-14-98

**Published:** 2014-12-11

**Authors:** Kelvin A Moses, Ling Y Chen, Daniel D Sjoberg, Melanie Bernstein, Karim A Touijer

**Affiliations:** Urology Service, Department of Surgery, Memorial Sloan-Kettering Cancer Center, 353 East 68th Street, New York, NY 10065 USA; Department of Epidemiology and Biostatistics, Memorial Sloan-Kettering Cancer Center, 307 East 63rd Street, New York, NY 10065 USA; Department of Urologic Surgery, Vanderbilt University Medical Center, 1161 21st Ave, MCN A-1302, Nashville, TN 37232 USA

**Keywords:** Prostate cancer, Radical prostatectomy, Race, Biochemical recurrence, Disparities, Age

## Abstract

**Background:**

Black men with prostate cancer are diagnosed at a younger age, present with more aggressive disease, and experience higher mortality. We sought to assess pathological features and biochemical recurrence (BCR) in young men undergoing radical prostatectomy (RP) to determine if there is a difference between black and white men closer to the time of disease initiation.

**Methods:**

We identified 551 white and 99 black men at a tertiary cancer center who underwent RP at ≤50 years of age. Baseline and pathological features were compared between the two groups. Cox proportional hazards models were utilized to examine the association of race and BCR, and Kaplan-Meier curves were generated to determine biochemical recurrence-free survival (bRFS).

**Results:**

There were no differences in median age at surgery, biopsy Gleason score, or comorbidity. Black men had higher preoperative PSA (6.1 ng/ml vs 4.7 ng/ml, p = 0.004), but a greater percentage were cT1c (78% vs 63%), compared to white men. On multivariate analysis, black men demonstrated significantly lower odds of non-organ confined disease (OR 0.39; 95% CI: 0.18, 0.81; p = 0.01) and extracapsular extension (ECE) (OR 0.38; 95% CI: 0.18, 0.81, p = 0.01), and had no difference in Gleason score upgrading and seminal vesicle invasion compared to white men. There was no significant difference in bRFS in men with organ-confined disease; however, among men with locally advanced disease black men trended towards greater BCR (p = 0.052). Black men had 2-year bRFS of 56% vs 75% in white men.

**Conclusions:**

In this single institution study, there does not appear to be a racial disparity in outcomes among younger men who receive RP for prostate cancer. Black and white men in our cohort demonstrate similar bRFS with pathologically confirmed organ-confined disease. There may be greater risk of BCR among black men locally advanced disease compared to white men, suggesting that locally advanced disease is biologically more aggressive in black men.

## Background

Black men have the highest incidence of prostate cancer and a 2.4-times greater mortality from prostate cancer compared to white men in the US [1]. Additionally, prostate cancer in black men tends to present at a younger age with more adverse pathological characteristics such as higher Gleason scores, greater tumor volume, and advanced disease [2–5]. A significant focus of research into this disparity is to identify the potential source(s). One potential source may be that differences in treatment received by black men may play a role in poorer outcomes. Black men are less likely to receive definitive therapy (surgery or radiation) vs androgen deprivation therapy and are less likely to receive surgery, regardless of stage at presentation [6–9]. It is unknown if treatment choice is more influenced by patient or physician factors, though both likely play a significant role [10–12]. Nevertheless, several studies have shown improved outcomes in black men that receive RP in terms of BCR and disease-free survival [5, 13, 14].

A second hypothesis to explain the mortality disparity is that prostate cancer biological behavior differs in black men compared to white men. Sanchez-Ortiz et al. showed that among men who underwent RP with cT1c disease and similar biopsy characteristics, black men have greater tumor volume, higher Gleason scores, and nearly 3 times more tumor per ng/ml of serum PSA [15]. Similarly, other groups have shown greater tumor volumes in black men compared to white men with similar clinical characteristics [16, 17] which potentially could translate to greater risk of BCR and disease-free survival [18].

Third, it has been postulated black men present with later stage disease, and thus are at increased risk for prostate cancer mortality. Some reasons for later stage at presentation include lack of insurance [19], less frequent or absent pre-diagnosis PSA testing [20], faster growth rate of cancer from the time of initial disease [17], and higher rates of obesity [21]. However, data indicate that the overall stage shift seen in recent years is now being observed in black men, which may serve to improve survival outcomes [14, 22].

We sought to compare the clinical features and rate of BCR of men undergoing RP in a tertiary care center. We analyzed men prior to age 50 to determine if differences in prostate cancer behavior nearer to the time of initial disease lead to poorer outcomes in black men. Preoperative PSA and stage, pathological features and the rate of BCR were assessed, and bRFS was compared between black and white men. We hypothesized that younger black men with localized disease who receive RP will achieve similar outcomes to white men.

## Methods

After obtaining Memorial Sloan-Kettering Cancer Center Institutional Review Board approval to access patient data, we identified 741 prostate cancer patients aged 50 or less who self-identify as “black” or “white” race and underwent a RP at MSKCC. Eighty-nine patients with surgery dates prior to year 2000 with incomplete data were excluded from the analysis. Two patients with neoadjuvant hormone and radiation therapy were also excluded from the analysis. The remaining 650 patients constituted the study cohort.

The primary aim is to compare differences in adverse pathological features between black and white men 50 years and younger. The age of 50 years was chosen as the cutoff because, although some guidelines now recommend earlier screening in black men, during the time period that comprised this study this was the age at which men are recommended to start PSA screening. For univariate analysis, we utilized the t-test for continuous variables, and Chi-square analysis for categorical variables. We used logistic regression models to assess whether black men have a higher probability of adverse pathological features (non-organ confined disease, seminal vesicle invasion (SVI), ECE, upgrade in Gleason score between biopsy and pathology) than white men. An upgrade in Gleason score was defined as an initial score of 6 or lower to 7 or higher on pathology or an initial score of 7 to 8 or higher on pathology. Biopsy Gleason scores of 8 were excluded from the model predicting upgrade in Gleason score as there is no higher Gleason category. The adverse outcomes were examined separately in logistic regression models adjusted for race along with important preoperative characteristics of the tumor including preoperative PSA, biopsy Gleason score, and clinical stage. As such, our question specifically pertains to whether black men have more aggressive tumors. No patients with biopsy Gleason grades of 6 or lower had lymph node involvement (LNI), and they were subsequently excluded from that analysis. Additionally, because so few patients had LNI, this analysis was limited to the univariate setting.

We conducted additional analyses to explore if race was associated with biochemical recurrence using Cox proportional hazards regression models. We evaluated the association between race and BCR adjusting for preoperative PSA, biopsy Gleason score, and clinical stage. A separate model was created adjusting for preoperative PSA, pathologic Gleason score and pathologic stage (organ- confined vs. locally advanced). bRFS curves were calculated separately for organ- confined and locally advanced disease using Kaplan-Meier methods and differences in survival times compared using the log –rank test. All statistical analyses were conducted using STATA 11.0 (Stata Corp., College Station, TX, USA).

## Results

Table 1 summarizes baseline and pathologic characteristics of 99 black and 551 white patients under 50 who underwent RP between January 2000 and February 2011. There was a significant difference between the two groups in terms of preoperative clinical stage, with black men having a higher percentage of cT1c disease compared to white men. Black men did have significantly higher preoperative PSA (6.1 ng/ml vs. 4.7 ng/ml, p = 0.004)), and a greater percentage of Black men had CCI >2; however, there were no differences in median age at surgery, or biopsy Gleason grade. There were no significant differences in overall pathological characteristics between the two groups in terms of Gleason score, extracapsular extension, lymph node involvement, or seminal vesicle invasion.

**Table 1:** **Preoperative and pathologic characteristics of men younger than 50 undergoing RP (n = 650)**

	Black N = 99	White N = 551	p-value
Preoperative characteristics			
Age at surgery	47 (45, 48)	47 (45, 49)	0.08
Preoperative PSA	6.1 (4.0, 8.3)	4.7 (3.0, 6.7)	0.004
Biopsy Gleason score			0.3
≤6	51 (53%)	329 (61%)	
7	38 (40%)	177 (33%)	
≥8	7 (7%)	31 (6%)	
Clinical Stage			0.03
T0, T1a, T1b	1 (1%)	3 (1%)	
T1c	76 (78%)	347 (63%)	
T2a	11 (11%)	113 (21%)	
T2b	7 (7%)	45 (8%)	
≥T2c	2 (2%)	31 (7%)	
Charlson comorbidity score			0.04
≤2	5 (5%)	20 (4%)	
3-5	73 (74%)	463 (84%)	
≥6	21 (21%)	68 (12%)	
**Pathologic characteristics**			
Pathologic Gleason score			0.6
≤6	35 (35%)	219 (40%)	
7	58 (59%)	294 (54%)	
≥8	6 (6%)	30 (6%)	
Extracapsular extension	14 (14%)	124 (23%)	0.06
Lymph node involvement			0.4
Positive	2 (2%)	28 (5%)	
Negative	88 (89%)	483 (88%)	
Not performed	9 (9%)	40 (7%)	
Seminal vesicle invasion	5 (5%)	25 (5%)	0.8

All values are median (IQR) or frequency (proportion).

After adjusting for preoperative PSA, biopsy Gleason grade, and clinical stage, black race had a highly protective effect for non-organ confined prostate cancer in men under 50 (Table 2). The odds of non-organ confined disease at the time of RP for black race was 0.39 (95% CI: 0.18, 0.81 p = 0.01), in other words, among men under 50, black race was associated with a statistically significant 60% reduction in the odds of non-organ confined disease for a given stage, grade, and PSA. When non-organ confined disease was stratified by ECE, SVI, and LNI, we found black race was only significantly associated with lower odds of ECE (OR 0.38, 95% CI: 0.18, 0.81, p = 0.01). There was no significant association between race and SVI or LNI among younger men (p = 0.9 and p = 0.16 respectively). The 95% confidence intervals for both LNI and SVI include the estimate for non-organ confined disease, so we do not see evidence of a differential effect of race on SVI versus LNI versus ECE. Black race also did not have a significant effect on upgrade in Gleason score in men under 50 (OR 0.97, 95% CI: 0.54, 1.74 p = 0.9).

**Table 2:** **OR of adverse pathological findings for black race (vs. white race)**

Adverse pathological outcome	OR	95% CI	P-value
Non organ confined disease	0.39	0.18,0.81	0.01
Extracapsular extension	0.38	0.18,0.81	0.01
Seminal vesicle invasion	0.93	0.30,2.93	0.9
Lymph node involvement*	0.36	0.08,1.53	0.16
Upgrade in Gleason score	0.97	0.54,1.74	0.9

Adjusted for preoperative PSA, biopsy Gleason grade, and clinical stage.

*Univariate analysis in biopsy Gleason scores of 7 or more only.

Given the significant association between race and pathologic stage, we explored recurrence outcomes between black and white patients. At last follow up, 59 patients recurred and median follow up in patients without biochemical recurrence was 3.5 years. After adjusting for preoperative variables, there was no evidence that race was significantly associated with bRFS (p = 0.9).

**Figure 1:**
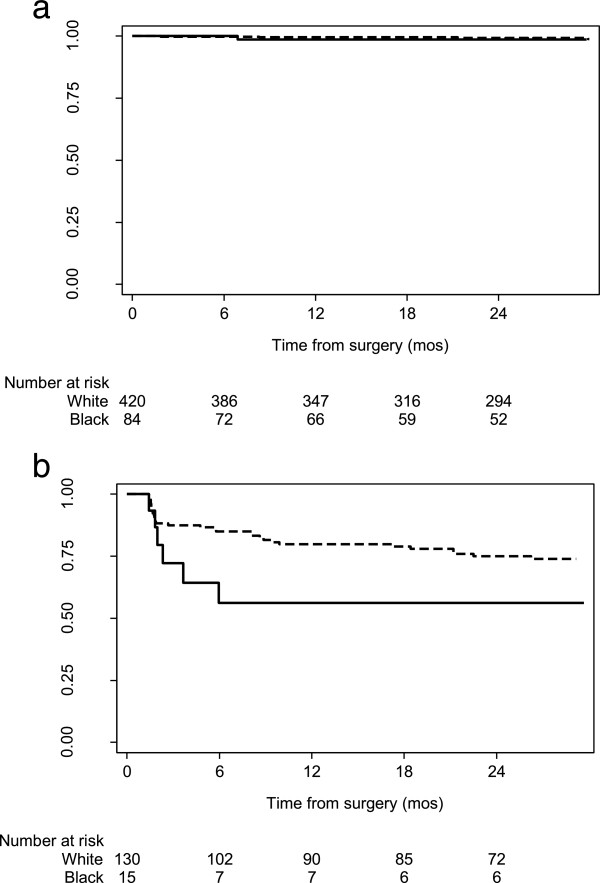
**Kaplan-Meier curve for bRFS in patients with a) organ confined disease (p = 0.4), and b) locally advanced disease (p = 0.052).** (Black = solid, White = dashed).

We conducted Kaplan-Meier analyses to investigate if a survival difference among black and white patients is apparent in both organ-confined and locally advanced disease. There was no statistically significant difference in bRFS between white and black patients with organ-confined disease (p = 0.4 by the log-rank test, Figure 1a). Kaplan Meier survival curves demonstrate black patients with locally advanced disease were at a greater risk of an earlier BCR than white patients (Figure 1b) and this result was close to statistical significance (p = 0.052 by log-rank test). This was also a small sample of black patients with 7 recurrences among 15 black patients with locally advanced disease. The 2-year bRFS was 56% (95% CI 27%, 78%) among patients of black race and 75% (95% CI 66%, 82%) among patients of white race.

## Discussion

In this study of men younger than 50 years of age undergoing RP for clinically localized prostate cancer, we show that black race is associated with lower odds of non-organ confined disease and ECE. This finding may not necessarily be a biological effect, but potentially a reflection of more aggressive screening of black men in this cohort. This is demonstrated by the difference in presentation of cT1c disease (77% black vs 63% white), and a larger proportion of black men having lower sub-classification of T2a and T2b than white men (90% vs 94%). Our findings are in keeping with data from others showing favorable outcomes in younger men undergoing RP [3]. We also show that while black and white men with localized disease have no difference in BCR, black men with locally advanced disease have reduced bRFS (56% vs 75%).

A significant proportion of black and white men in our cohort presented with cT1c disease. The use of PSA screening and refinement of guidelines has led to a significant stage shift over time in all men, though black men have lagged in the percentage of men diagnosed with regional or distant disease [23]. Bianco et al. reported an improvement in clinical stage, preoperative PSA and biopsy Gleason score in men with prostate cancer treated after 1996 [14]. Black men in the group treated after 1996 had no significant difference in cancer recurrence-free survival compared to white men, and demonstrated a 20% increase in disease-free survival. In contrast, prior to 1996, black race was an independent predictor for pathological Gleason score and stage. Therefore, black men have derived a documented survival benefit by the stage shift seen in the PSA era. Further evidence that appropriate screening can improve clinical stage at presentation comes from Jones et al. who showed a significant reduction in the association of black race and risk of advanced disease in men who received digital rectal examination and/or PSA testing [24]. The risk of advanced disease was not completely mitigated with PSA and digital rectal examination, nor was it when controlling for other sociodemographic factors, thus indicating a difference in the biological behavior of prostate cancer in black men.

Our study demonstrates that black men had similar or better pathologic outcomes compared to white men younger than 50 when receiving RP. Other groups have demonstrated improved outcomes in black men who have RP as their primary mode of treatment. In a study of 2407 men following RP for low-risk prostate cancer, Resnick et al. found no significant differences in BCR, locally advanced disease, or risk of disease upgrading between black and white men [13]. The results were not stratified by age so it is unknown whether men younger than 50 demonstrate similar results, but their findings suggest that black men with low risk disease who have RP can achieve equivalent results to white men. Underwood et al. showed similar cancer-free survival between black and white men who had RP, when matched for stage and grade [25]. Black men in their overall cohort were significantly younger, which suggests a potential benefit to earlier screening and aggressive treatment as a way of reducing the mortality disparity. These results are encouraging; however, black men are less likely to have RP as the primary treatment modality and more likely to have RT or ADT than whites with similar stage and grade [6, 26].

We show that black men with locally advanced disease may be at greater risk for BCR than white men who have received the same treatment, suggesting that black men may display more biologically aggressive tumors at higher stages. In a study of men undergoing RP at Wayne State University, Wood et al. showed no difference in the rate of organ-confined disease after RP between black and white men (76.3% vs 71.0%) [27]. However, they reported a 12% lower absolute difference in bRFS 5 years after RP in black men despite a significant shift in disease characteristics at presentation. This difference in bRFS was similar to that seen earlier in the PSA era (11%). Their data suggest aggressive biological behavior influences long-term outcomes, particularly in higher stage patients. This is confirmed in their analysis of 612 patients who underwent RP between 1991 and 1995, comparing the rate of BCR between black and white men at a mean of 34 months follow-up [28]. Similar to the findings of this study, the authors found that black men with locally advanced disease were more likely to experience BCR, but not those with organ-confined disease. They also found a trend towards a greater percentage of high grade Gleason patterns in black men. Using data from their institution and SEER, Powell et al. have suggested that although tumor characteristics at disease initiation in black and white men are similar, black men have faster growing tumors and progress to advanced or metastatic disease earlier in the course of the disease [17]. In fact, black men demonstrated advanced or metastatic disease at a 4:1 ratio compared to white men. This may explain why black men with organ-confined disease have similar postoperative outcomes to white men, as the disease is likely closer to that seen at initiation. However, faster growth/transformation is clearly recognized in black men, which has led to a search for genetic variants or other molecular markers for aggressive disease in men of different ethnic backgrounds. Recent studies have yielded some clues to a biological basis of aggressive tumor features. Haiman et al. performed a genome wide study in men of African ancestry and found a significant association with a variant of chromosome 17q21 in prostate cancer cases rarely seen in men of other populations [29]. Other potential genetic targets, such as 11q22 and Xq21, have been identified by genome-wide linkage analysis in the African-American Hereditary Prostate Cancer study [30]. The use of this large study of African-American families with at least four affected member will be critical to identify targets for risk of prostate cancer, and potentially may help predict risk for aggressive prostate cancer growth.

Our results are to be interpreted in the context of some limitations. MSKCC is a tertiary/quaternary referral center, so results from our cohort are not necessarily applicable to the general population. In particular, black men who receive treatment are likely to have higher socioeconomic status compared to other studies completed in underinsured/uninsured populations, and likely were more aggressively screened than men with lower socioeconomic status. Additionally, the overall number of patients is relatively small, especially among those who experienced BCR. The a priori age limit we set for the purposes of this study restricts the numbers of patients available for study, which is not likely to be different than other institutions that treat large numbers of patients. However, our results are bolstered by the ability to compare a relatively similar cohort of black and white men, which strengthens the conclusions we make regarding the improved outcomes of black men who receive RP.

## Conclusions

We show that younger black men who receive RP for clinically localized prostate cancer can achieve similar outcomes compared to white men. Black men were at no additional risk of Gleason upgrading compared to young white men. Although we demonstrate an association of black race with lower odds of having locally advanced disease, these findings likely reflect the benefit of aggressive screening and treatment. Importantly, we show that black men with pathologically locally advanced disease may be at higher risk for biochemical recurrence than white men after RP. These results suggest that progression to later stage disease poses an increased risk for poorer outcomes in black men. Future studies on early screening in black men combined with aggressive definitive therapy should be conducted to assess the impact of stage at presentation on BCR and survival. Additionally, in men with locally advanced disease, comparison of specific genetic targets may explain differences in the biological behavior of prostate cancer in black men.

### Consent

Written informed consent was obtained from the patient for the publication of this report and any accompanying images.

## Abbreviations

ADT: Androgen deprivation therapy
BC: Biochemical recurrence
bRFS: Biochemical recurrence-free survival
ECE: Extracapsular extension
LNI: Lymph node invasion
MSKCC: Memorial Sloan-Kettering Cancer Center
PSA: Prostate-specific antigen
RP: Radical prostatectomy
RT: Radiation therapy
SVI: Seminal vesicle invasion.
